# Comparison between the protective effect of the orally administered atorvastatin and safflower (*Carthamus tinctorius)* in hypercholesterolemic male rats

**DOI:** 10.3389/fphar.2025.1663717

**Published:** 2025-09-15

**Authors:** Haddad A. El Rabey, Eman S. Attia, Nadia Bakry, Samar M. Rezk, Asmaa Y. Sharfeldin

**Affiliations:** ^1^ Biochemistry Department, Faculty of Science, University of Tabuk, Tabuk, Saudi Arabia; ^2^ School of International Education, Hainan College of Economics and Business, Hainan, China; ^3^ National Nutrition Institute, Egypt Ministry of Health and populations, Cairo, Egypt; ^4^ Bone Marrow Transplantation and Cord Blood Unit, Mansoura University, Mansoura, Egypt; ^5^ Clinical Nutrition Department, Mahalla Hepatology Teaching Hospital, Egypt Ministry of Health and Populations, El-Mahalla El-Kubra, Gharbyia, Egypt; ^6^ Department of Public Health and Community Medicine, Faculty of Medicine, Menoufia University, Shebin El Kom, Egypt

**Keywords:** adrenaline, atorvastatin, hypercholesterolemia, safflower, troponin, vitamin D

## Abstract

Hyperlipidemia is correlated with the elevation of cholesterol and triglyceride levels in the blood that increase the risk of cardiovascular events, such as heart attacks and strokes. This study aimed to test the hypolipidemic activity and other health benefits of atorvastatin and safflower (*Carthamus tinctorius* L., family *Asteraceae*) on rats with induced hypercholesterolemia in a four-week study. 24 male albino rats were divided into four groups (n = 6). The first group (G1) was given a normal basal diet as a negative control, while the other rats received a high-fat diet with 5% cholesterol. The second group (G2) served as the positive control, receiving no treatment. The third group (G3) received 200 mg/kg body weight safflower aqueous extract, and the 4th group (G4) received 20 mg/kg body weight atorvastatin. The induced hypercholesterolemia significantly raised liver function enzymes, lipid peroxidation (14.9 ± 0.11 mg/dL), total cholesterol (273.3 ± 1.1 mg/dL), triglycerides (223.0 ± 4.1 mg/dL), low-density lipoproteins (204.7 ± 0.9 mg/dL), very low-density lipoproteins (44.6 ± 0.8 mg/dL), troponin, creatine kinase (CK), and adrenaline while decreased antioxidant enzymes, high-density lipoprotein (HDL), and vitamin D (11.1 ± 0.5 ng/mL). The liver and heart tissues were also significantly injured by hypercholesterolemia. Administration of atorvastatin and safflower markedly ameliorated the biochemical and histological abnormalities associated with induced hyperlipidemia, restoring them to near-normal levels. Atorvastatin treatment in G4 demonstrated superior efficacy compared to safflower extract in addressing hypercholesterolemia, despite the latter’s significant hypolipidemic effect observed in G3.

## 1 Introduction

Hypercholesterolemia or dyslipidemia occurs when low-density lipoprotein (LDL) rises and accumulates fat in the arteries (atherosclerosis) and cardiovascular diseases, increasing the risk of heart attacks and contributing to more deaths (El Rabey et al., 2018; Guo et al., 2022; Li et al., 2023). It is a severe health issue that impacts the body’s oxidative stress, rendering it vulnerable to a variety of illnesses, including Alzheimer’s, osteoporosis, diabetes, high blood pressure, cancer, obesity, and aging (Al-Sieni et al., 2020; Jomova, et al., 2023; Li et al., 2023).

In addition, hyperlipidemia induces oxidative stress through the buildup and oxidation of low-density lipoproteins (LDL) cholesterol in artery walls, which leads to inflammation and ultimately artery narrowing that, in turn, raises the risk of heart attacks, strokes, and cardiovascular diseases (Zmysłowski and Szterk, 2017; Prasad and Mishra, 2022). In addition, the World Health Organization (WHO) estimates that approximately 39% of people worldwide have high cholesterol (Sawhney and Madan, 2024). The frequency of familial hypercholesterolemia (FH) in the general population is estimated to be between 1 in 250 to 1 in 311 (Vaezi and Amini, 2025). Regional variations exist, with founder effects leading to a higher incidence in specific populations in the Middle East, Quebec, and South Africa (Vaezi and Amini, 2025; Sawhney and Madan, 2024).

Chemical-based medicines may cause adverse reactions, such as liver problems, nausea, drowsiness, diarrhea, or allergic reactions, known as an adverse drug reaction (ADR) (Kommu et al., 2025; Coleman and Pontefract, 2016). To avoid the negative effects of chemical medicine, people all over the world prefer to use herbal medicine to treat and prevent hypercholesterolemia. This has led scientists to investigate the benefits of employing herbal remedies and natural products to reduce hypercholesterolemia, lessen the risk of cardiovascular disorders, and shield the heart from heart attacks (El Rabey et al., 2018; Al-Sieni et al., 2020; Alshareef et al., 2024).

The safflower (*Carthamus tinctorius* L.; Family *Asteraceae*) is a highly branched annual herbaceous plant grown for its culinary and medicinal properties (Delshad et al., 2018; Adamska and Biernacka, 2021). Its yellowish-red blossoms are used for many medical purposes, and its seeds are crushed to yield edible oil. Because of its analgesic and antipyretic properties, it has also been used in traditional medicine to alleviate the symptoms of menstrual discomfort. It was also used in food coloring and flavoring as well as other medicinal applications such as a purgative, an antidote for poisoning, and as a remedy for postpartum hemorrhage, trauma, joint discomfort, and osteoporosis (Delshad et al., 2018).

Worldwide, safflower seeds have long been used to prevent osteoporosis and encourage bone growth (Che et al., 2016). Carthamin, safflower yellow, safflamin C, isocarthamidin, hydroxysafflor yellow A, Carthamidin, safflor yellow A, luteolin caryophyllene, 1-acetoxytetralin, heneicosane, and p-allyltoluene are the primary active ingredients of safflower flowers (Delshad et al., 2018). According to Adamska and Biernacka (2021), the primary ingredient in safflower flower extract, hydroxysafflor (HSYA), exhibits anti-inflammatory, antibacterial, and antioxidant properties, and has been shown to combat obesity in rats and mice.

The FDA-approved medication atorvastatin, often known as Lipitor, is a synthetic drug of the statin class used to treat high blood pressure and heart issues by preventing cardiovascular illnesses in patients with dyslipidemia and cardiac risk factors (Livingstone et al., 2016; Abd-Elmonsif and Gamal, 2025).

Vitamin D is a micronutrient, sunlight-dependent vitamin that maintains a healthy skeleton and guards against liver fibrosis and is considered vital for numerous metabolic processes (Che et al., 2016; Keane et al., 2018). Rickets, osteomalacia, cancer, and cardiovascular disease all occur due to a lack of vitamin D, which is either produced endogenously as vitamin D3 (cholecalciferol) or acquired through diet (ergocalciferol) (Zhang et al., 2017; Deepika et al., 2025). Numerous diseases, including inflammation, carcinogenesis, endocrinopathies, autoimmunity, type 1 and type 2 diabetes mellitus, Hashimoto’s or Graves’ thyroiditis, Cushing’s disease, hyperaldosteronism, adrenocortical tumors, and the adrenal gland, have been linked to vitamin D, according to both *in vitro* and *in vivo* research (Al Refaie et al., 2022).

As a chemical messenger that serves as a central nervous system neurotransmitter to convey nerve impulses within nerve, muscle, or gland cells, adrenaline, also known as epinephrine, is generated by the adrenal glands and is “a part of the emergency response system to the fight-or-flight response.” Costa and Schoenbaum, 2022). Sugar consumption and hypercholesterolemia cause metabolic dysfunction as well as other mental and neurological conditions such as anxiety and depression (Fulton et al., 2022).

This study was focused at clarifying the probable hypolipidemic effect of atorvastatin and safflower in male rats with induced hypercholesterolemia. Some other therapeutic activities of both materials were also studied.

## 2 Materials and methods

### 2.1 Chemicals and test materials

The safflower flowers were purchased from a spice store in Mansoura City, Egypt. The safflower flowers were identified by Dr Yasser El-Amier - A plant taxonomist at Botany Department, Faculty of Science, Mansoura University- as *Carthamus tinctorius* L. and a specimen with accession number “Mans.0161615641” was deposited in the Herbarium of the Botany Department, Faculty of Science, Mansoura University, Egypt. Atorvastatin was purchased from a nearby pharmacy with the commercial name “Ator”. The other chemicals were purchased from Sigma-Egypt and were of molecular biology grade. The kits in question were acquired from the appropriate vendors.

### 2.2 Test diet

The basal diet Ain-93, developed by the American Institute of Nutrition (AIN), was purchased from a local rodent care shop in Mansoura City, Egypt. The diet contains a balanced combination of casein, soybean oil, cellulose, cornstarch, a mineral mix, sucrose, and a vitamin mix. A 5% cholesterol was added to the basal diet for preparing the high-fat diet (Oyetayo et al., 2020). Cholesterol powder (5%w/w) was well combined with crushed pellet diet; the pellets were then reconstituted with water and adequately dried to prevent fungal contamination.

### 2.3 Test animals and work plan

A 7-week-old male Sprague-Dawley albino rats (n = 24, weight: 172.3 ± 0.6 g) were obtained from the Agricultural Research Center in Giza, Egypt as lab experimental animals for this study. Rats were kept in stainless steel cages (6/each) and monitored for a week before the experiment’s commencement in a controlled environment with consistent lighting (12 h light/12 h dark cycle), 30%–40% humidity and 25 °C room temperature. On 30-09-2024, the Ethical Committee of the Faculty of Pharmacy at Mansoura University approved the study plan (Key: MD-ACUC-SC.R.24.09.18), and the experiment was conducted for 4 weeks based on this approved study plan. The rats were divided into four groups (n = 6) as follows: the first group (G1), as a negative control, received 1 mL of distilled water by gavage and fed a basal rodent diet. To induce hypercholesterolemia, the other 18 rats were divided into three groups and received a fat-rich diet with 5% cholesterol (Oyetayo et al., 2020). As the positive control group, the second group received only 1 mL of distilled water by gavage, the third group received a daily dose of safflower extract via stomach gavage at a dose of 200 mg/kg body weight (Karami et al., 2017), and the fourth group (G4) received a daily oral dose of 20 mg/kg body weight atorvastatin dissolved in distilled water (Jabir and Jaffat, 2018). Feed and water were provided *ad libitum*.

### 2.4 Safflower aqueous extract preparation

For daily treatment, 5 g of the powdered flowers are macerated in 100 mL of heated distilled water for 1 h until they cool, and then used (Karami et al., 2017).

### 2.5 HPLC analysis of safflower aqueous extract

The polyphenols of safflower aqueous extract were determined using the HPLC system at the National Research Center (NRC) in Giza, Egypt, according to UV and diode array detection (DAD) methods (Mizzi et al., 2020).

### 2.6 Collection of blood and samples

After the experiment period (4 weeks) and following a 12-h fast, the rats were euthanized by CO_2_ (El Rabey et al., 2023) as follows: rats were exposed to CO_2_ flow by displacing 30%–70% of the cage volume per minute until narcosis without removing them from their home to avoid stress caused by handling and then the narcotic rats were subjected to cervical dislocation. Following euthanasia, rats were dissected, blood was drawn from the heart, and then placed in a plain tube and stored in a fridge at 4 °C for biochemical examination within 1 week. For histological preparations, a piece of the liver, a portion of the heart, and one kidney were preserved in 10% formalin. To prepare the homogenate, a portion of the liver was rinsed with saline and placed on ice. All procedures were conducted under aseptic conditions.

### 2.7 Preparation of liver homogenate

To estimate the lipid peroxidation and antioxidant activity, the liver tissue homogenate was prepared according to El Rabey et al. (2020). Briefly, a piece of ice-cold liver tissue was cut into small pieces, rinsed in cold phosphate buffer, homogenized, sonicated for 15 s, and then centrifuged under cooling (4 °C) at 12,000 rpm for 5 min. The resulting supernatant was kept in the fridge until use for the determination of antioxidants and lipid peroxidation.

### 2.8 Biochemical evaluations

#### 2.8.1 Liver parameters

The BSM Bioscience medical kits, Madrid (Spain), were used to estimate alkaline phosphatase (ALP), aspartate transaminase (AST), and alanine aminotransferase (ALT) using an optimized IFCC. Enzymatic – UV method (Cat No. 70108 for ALP, 70110 for AST, and 70111 for ALT). The BIOBASE kits (Jinan, China) were used to assess the total protein (TP), albumin, and bilirubin. The TP was estimated using the Biuret method (Cat. No: 304L), albumin was determined using the Bromcresol Green method (Cat. No.: 424L), and bilirubin was estimated according to the Vanadate oxidation method (Cat. number: 430L).

#### 2.8.2 Parameters of the lipid profile

The BT kits from Rome (Italy) were used to estimate triglycerides (TG), total cholesterol (TC), high-density lipoproteins (HDL), and low-density lipoproteins (LDL) using the enzymatic colorimetric GPO-PAP method (Cat. No.: 315L for TG, 135L for TC, 143L for HDL, and 145L for LDL). Furthermore, the very low-density lipoprotein (vLDL) was 1/5 of the triglycerides.

#### 2.8.3 Lipid peroxidation and antioxidants

Following the supplier’s instructions, the activities of catalase (Cat), glutathione-s-transferase (GST), and superoxide dismutase (SOD) were measured spectrophotometrically using the Biodiagnostic Kit (Cat. No.: GR 25 11), Cairo (Egypt). The Biodiagnostic Kit was also used to assay the lipid peroxidation product, malondialdehyde (MDA), in the liver tissue homogenate according to the supplier’s instructions.

#### 2.8.4 Estimation of troponin and creatine kinase (CK)

Rat Troponin I ELISA Kit (Cat. No.: MBS765393) from ABCAM, Cambridge (UK) was used to estimate the heart serum troponin, and the MG KIT (Cat No.: MBS269244) of My BioSource, San Diego (USA) was used to quantify creatine kinase (CK).

#### 2.8.5 Evaluation of vitamin D (Vit D) and adrenaline

Vit D was also determined by ELISA using My BioSource San Diego (USA) Rat kit (1,25-Dihydroxyvitamin D3, Calcitriol) ELISA Kit (Cat. No.: MBS2601701. The Rat Epinephrine, EPI ELISA Kit (Cat. No.: EA0043Ra), Shanghai (China), was used for serum adrenaline estimation. All analyses were conducted as described in the supplier’s guidelines.

#### 2.8.6 Serum Ca^++^ level estimation

The Bioteciva kit and the Bioteciva instrument S.P.A. 1600 Rome (Italy) were used to estimate the serum levels of Ca ions (Cat. No.: 432L).

### 2.9 Histopathological investigations

4–5 μm thin sections of both liver and heart were prepared from the 10% formalin fixed samples (after ethanol dehydration, xylene clearance, and embedding in paraffin), stained with hematoxylin and eosin dye, and finally examined using an Olympus light microscope with a digital camera (Gurina and Simms, 2025).

### 2.10 Analysis of statistics

To determine the Mean ± SE and the t-test values (∗∗∗ *P* < 0.001: extremely significant and ∗∗ *P* < 0.05: significant), all data were examined using the SPSS software version 26 (SPSS Inc. Released 2018. IBM SPSS Statistics for Windows, version 26.0, Armonk, NY: IBM Corp.). Means with different superscripts (a, b, c, or d) indicate a significant difference at *P* < 0.05, whereas means with the same letters indicate no significant difference at *P* < 0.05. The analysis of variance (ANOVA) between groups was also computed. For significant ANOVA tests, the post-hoc test is the least significant difference (LSD).

## 3 Results

### 3.1 HPLC analysis of the safflower extract

15 polyphenols were obtained from the HPLC analysis of the polyphenols of the safflower extract as follows: quercetin was the highest constituent (52289.14 μg/g), chlorogenic acid (18761.89 μg/g), Syringic acid (5193.76 μg/g), Cinnamic acid (1923.21 76 μg/g), Gallic acid (1509.43 μg/g), Rosmarinic acid (1311.60 μg/g), Methyl gallate (943.14 μg/g), Naringenin (707.92 μg/g), Ellagic acid (591.76 μg/g), Daidzein (525.72 μg/g), Ferulic acid (507.30 μg/g), Vanillin (413.68 μg/g), Hesperetin (217.09 μg/g), Kaempferol (139.51 μg/g), and the lowest was coumaric acid (126.65 μg/g) (Supplementary Figure S1).

### 3.2 Effect of safflower and atorvastatin on lipid profile in induced hypercholesterolemic rats

Table 1 demonstrates that the induction of hypercholesterolemia in the positive control group (G2) led to a significant reduction in HDL and a significant elevation (*P* < 0.001) in total cholesterol, triglycerides, LDL, and VLDL compared to the negative control. Safflower extract significantly (*P* < 0.001) (*P* < 0.001) raised HDL and significantly (*P* < 0.001) decreased the altered lipid profile parameters (T. cholesterol, triglycerides, LDL, and VLDL) in the hypercholesterolemic mice in G3 compared to the positive control group (G2). In G4, atorvastatin exhibited stronger hypolipidemic action than safflower extract, despite the latter’s hypolipidemic effects.

**TABLE 1:** The hypolipidemic effect of safflower and atorvastatin administration on lipid profile in induced hypercholesterolemic male rats.

Variables	Statistics	G1 −ve control	G2 +ve control	G3 safflower	G4 Atorvastatin
T. cholesterol mg/dL	Mean ± SE LSD (0.05) = 5.81 t-test	156.3 ± 1.5^a^	273.3 ± 1.1^d^ −63.25***	227.7 ± 1.1^b^ −38.56***	202.3 ± 3.3^c^ −12.75 ***
TG mg/dL	Mean ± SE LSD (0.05) = 7.91 t-test	126.7 ± 2.0^a^	223.0 ± 4.1^d^ −21.24***	205.0 ± 0.7^b^ −36.61***	193.3 ± 2.8^c^ −19.50 ***
HDL mg/dL	Mean ± SE LSD (0.05) = 2.20 t-test	49.3 ± 0.9^a^	22.0 ± 0.9 20.50***	40.0 ± 0.4 9.44***	36.7 ± 0.6 11.78 ***
LDL mg/dL	Mean ± SE LSD (0.05) = 7.67 t-test	81.3 ± 1.8^a^	204.7 ± 0.9^d^ −60.02***	146.3 ± 0.6^b^ −33.85***	127.0 ± 4.7^c^ −8.97 ***
VLDL mg/dL	Mean ± SE LSD (0.05) = 1.58 t-test	25.3 ± 0.4^a^	44.6 ± 0.8^d^ −21.24***	41.0 ± 0.1^b^ −36.61***	38.7 ± 0.6^c^ −19.50 ***

Data are represented as Mean ± SE. t-test value “∗∗∗” means highly significant at *P* < 0.001. ANOVA analysis within groups: means with different superscripts (a, b, c, or d) show a significant difference at *P* < 0.05, while means with the same letters mean that there is no significant difference at *P* < 0.05. LSD: Least Significant Difference.

T. Cholesterol: Total cholesterol, TG, Triglycerides; HDL, High-density lipoprotein; LDL, Low-density lipoprotein; VLDL, Very Low-density lipoprotein.

### 3.3 Effect of safflower and atorvastatin on antioxidants and lipid peroxidation

**FIGURE 1:**
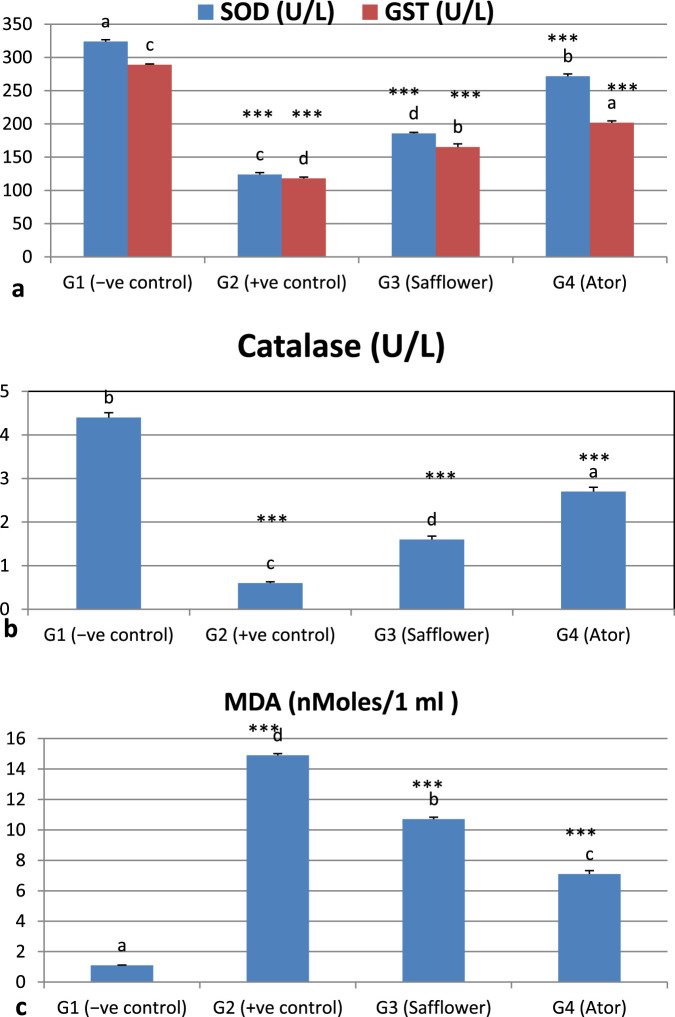
Effect of safflower and atorvastatin administration on antioxidants and lipid peroxidation of hypercholesterolemic male rats. **(a)** SOD and GST, **(b)** Catalase, **(c)** Lipid peroxidation (MDA). Different superscripts (a, b, c, or d) show a significant difference at *P* < 0.05. t-test values “∗∗∗” means highly significant at *P* < 0.001.

Figure 1 and Supplementary Table S1 indicate that the induction of hypercholesterolemia resulted in a significant (*P* < 0.001) reduction in the levels of antioxidant enzymes (SOD, catalase, and GST) and an increase in lipid peroxidation, as evidenced by MDA levels. Treatment of hypercholesterolemic male rats with safflower and atorvastatin in groups G3 and G4 resulted in a significant increase (*P* < 0.001) in antioxidant enzyme levels and a decrease in lipid peroxidation.

### 3.4 Effect of safflower and atorvastatin on liver function in induced hypercholesterolemic rats

**FIGURE 2:**
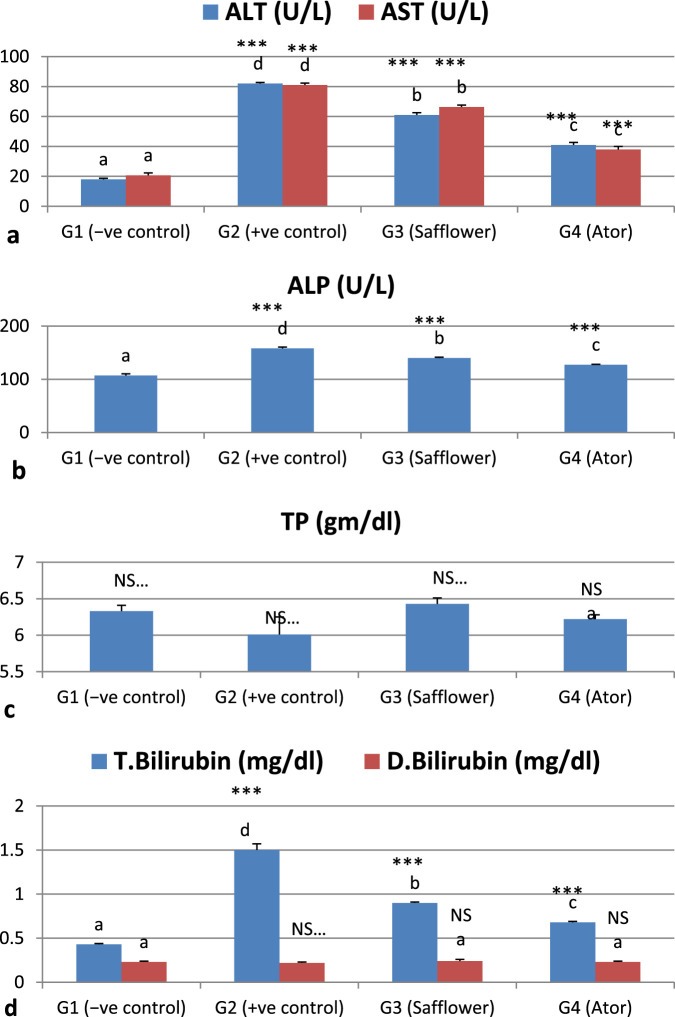
Effect of safflower and atorvastatin administration on liver function of hypercholesterolemic male rats. **(a)** ALT and AST, **(b)** ALP, **(c)** Total protein, and **(d)** Bilirubin (total and direct). Different superscripts (a, b, c, or d) show a significant difference at *P* < 0.05. t-test values “∗∗∗” means highly significant at *P* < 0.001, NS: non-significant.

The analyzed liver function markers, ALT, AST, ALP, and bilirubin, exhibited a significant (*P* < 0.001) increase due to the induced hypercholesterolemia, whereas the total proteins showed a non-significant increase, as illustrated in Figure 2 and Supplementary Table S2. The administration of safflower extract and atorvastatin to hypercholesterolemic rats in G3 and G4, respectively, significantly (*P* < 0.001) restored the altered parameters. Atorvastatin has shown superior efficacy in normalizing the abnormal liver function measures compared to safflower.

### 3.5 Effect of safflower and atorvastatin on heart function enzymes in induced hypercholesterolemic rats

**FIGURE 3:**
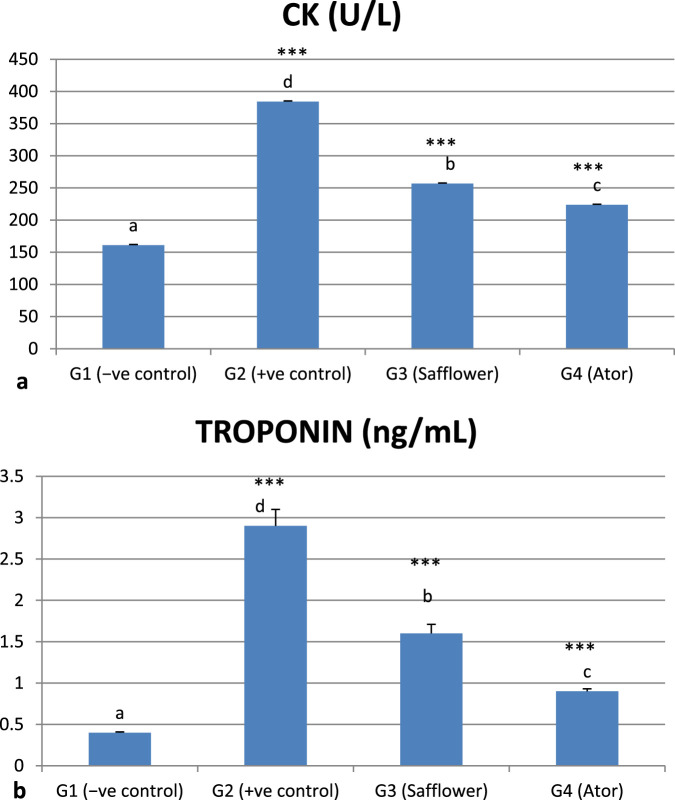
Effect of safflower and atorvastatin administration on heart function enzymes in hypercholesterolemic male rats. **(a)** CK, **(b)** Troponin. Different superscripts (a, b, c, or d) show a significant difference at *P* < 0.05. t-test values “∗∗∗” means highly significant at *P* < 0.001.

The induced hypercholesterolemia significantly increased the heart function enzymes, CK and Troponin, in G2 compared to the negative control G1 (*P* < 0.001), as illustrated in Figure 3 and Supplementary Table S3. Treatment of induced hypercholesterolemia in G3 and G4 with safflower and atorvastatin resulted in a significant decrease in elevated values (*P* < 0.001). Atorvastatin in G4 demonstrated stronger efficacy in improving heart function parameters compared to safflower.

### 3.6 Effect of safflower and atorvastatin administration on Vit D and C^++^ in hypercholesterolemic male rats

**FIGURE 4:**
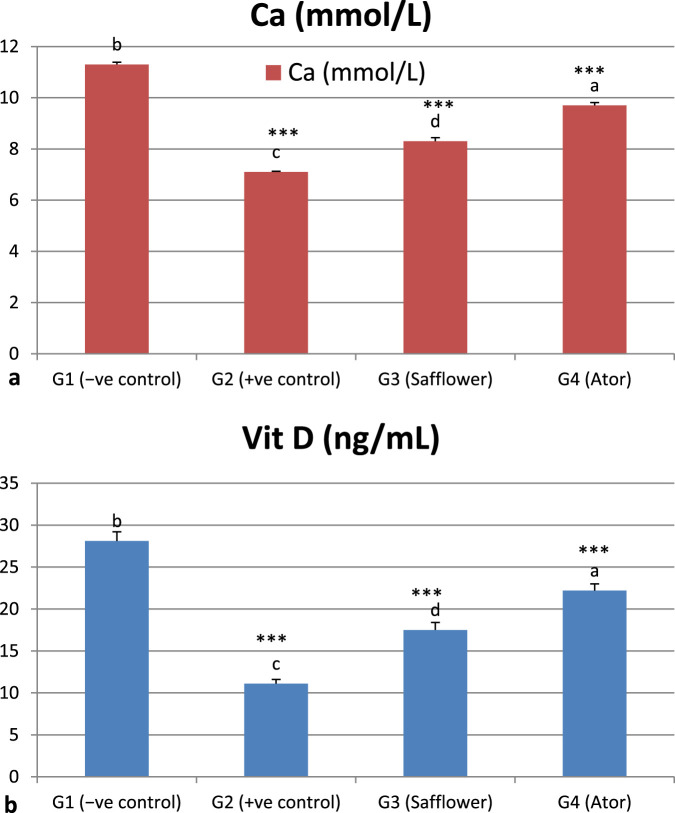
Effect of safflower and atorvastatin administration on calcium and vitamin D in hypercholesterolemic male rats. **(a)** Ca^++^, **(b)** Vit D. Different superscripts (a, b, c, or d) show significant difference at *P* < 0.05. t-test values “∗∗∗” means highly significant at *P* < 0.001.

Figure 4 and Supplementary Table S3 indicate that induced hypercholesterolemia resulted in a decrease in calcium ions, whereas treatment with safflower and atorvastatin led to an increase in their levels. Treating with atorvastatin was more efficient than safflower extract in G3. Similar to C++, vitamin D levels were significantly decreased (*P* < 0.001) due to hypercholesterolemia induction in G2, whereas treatment with safflower and atorvastatin in G3 and G4, respectively, increased their levels. Atorvastatin treatment resulted in higher levels of C++ and vitamin D in group G4 compared to safflower in group G3.

### 3.7 Effect of safflower and atorvastatin on serum adrenaline level

**FIGURE 5:**
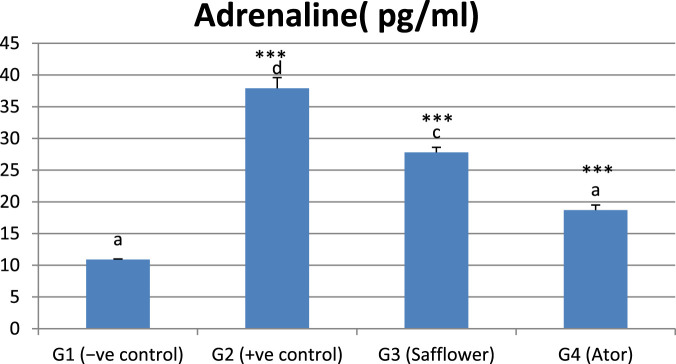
Effect of safflower and atorvastatin administration on adrenaline in hypercholesterolemic male rats. Different superscripts (a, b, c, or d) show significant difference at *P* < 0.05. t-test values “∗∗∗” means highly significant at *P* < 0.001.

Figure 5 and Supplementary Table S3 show that adrenaline was significantly (*P* < 0.001) increased by the induced hypercholesterolemia in G2 compared to the negative control (G1) and significantly (*P* < 0.001) decreased with safflower and atorvastatin treatment in G3 and G4, respectively. Atorvastatin in G4 demonstrated stronger efficacy in reducing adrenaline levels compared to safflower.

### 3.8 Correlation of the studied criteria

Table 2 presents the correlation among the various parameters examined. HDL is negatively correlated with total cholesterol, triglycerides, LDL, and VLDL. ALT, AST, ALP, and total bilirubin exhibited positive correlations with total cholesterol, triglycerides (TG), low-density lipoprotein (LDL), and very low-density lipoprotein (VLDL), while demonstrating negative correlations with high-density lipoprotein (HDL). Kidney function parameters, including creatinine, uric acid, and urea, exhibit correlations with total cholesterol, triglycerides, low-density lipoprotein, very low-density lipoprotein, and liver function parameters. Calcium ions exhibited a negative correlation with liver and kidney function parameters. Adrenalin, MDA, CK, and troponin exhibited positive correlations with elevated lipid and liver function parameters, while catalase, SOD, GSH, and vitamin D showed negative correlations.

**TABLE 2:** The correlation between different studied parameters.

Variables	T.cholesterol	TG	HDL	LDL	VLDL	ALT	AST	ALP	T.BILLI	D.BILLI	TP	Ca++	Adrenaline	Catalase	SOD	GST	MDA	TROPONIN	CK	VIT D
T.cholesterol		0.924**	−0.922**	0.995**	0.924**	0.988**	0.972**	0.955**	0.948**	−0.092-	−0.258	−0.976**	0.199	−0.977**	−0.968**	−0.972**	0.983**	0.963**	0.973**	−0.958-**
TG	0.924**		−0.860**	0.897**	1.000**	0.926**	0.891**	0.913**	0.798**	0.059	−0.295	−0.918**	0.24	−0.939**	−0.878**	−0.959**	0.945**	0.820**	0.839**	−0.868**
HDL	−0.922**	−0.860**		−0.944**	−0.860**	−0.890**	−0.826**	−0.876**	−0.894**	0.06	0.421*	0.865**	−0.056	0.863**	0.844**	0.878**	−0.891**	−0.894**	−0.946**	0.861**
LDL	0.995**	0.897**	−0.944**		0.897**	0.974**	0.952**	0.945**	0.962**	−0.113	−0.291	−0.961**	0.163	−0.959**	−0.950**	−0.952**	0.966**	0.967**	0.986**	−0.952-**
VLDL	0.924**	1.000**	−0.860**	0.897**		0.926**	0.891**	0.913**	0.798**	0.059	−0.295	−0.918**	0.24	−0.939**	−0.878**	−0.959**	0.945**	0.820**	0.839**	−0.868-**
ALT	0.988**	0.926**	−0.890**	0.974**	0.926**		0.979**	0.960**	0.933**	0.005	−0.235	−0.980**	0.208	−0.969**	−0.985**	−0.976**	0.987**	0.951**	0.957**	−0.939-**
AST	0.972**	0.891**	−0.826**	0.952**	0.891**	0.979**		0.930**	0.898**	−0.093	−0.181	−0.972**	0.268	−0.966**	−0.980**	−0.943**	0.962**	0.943**	0.919**	−0.946-**
ALP	0.955**	0.913**	−0.876-**	0.945**	0.913**	0.960**	0.930**		0.930**	0.1	−0.397	−0.941**	0.166	−0.962**	−0.943**	−0.968**	0.967**	0.925**	0.929**	−0.888-**
T.BILLI	0.948**	0.798**	−0.894**	0.962**	0.798**	0.933**	0.898**	0.930**		−0.060	−0.347	−0.920**	0.142	−0.905**	−0.928**	−0.910**	0.931**	0.967**	0.982**	−0.924**
D.BILLI	−0.092-	0.059	0.06	−0.113	0.059	0.005	−0.093	0.1	−0.060		−0.243	−0.025	0.211	0.006	−0.009	−0.033	0.034	−0.150	−0.070	0.211
TP	−0.258	−0.295	0.421*	−0.291	−0.295	−0.235	−0.181	−0.397	−0.347	−0.243		0.209	0.016	0.26	0.206	0.243	−0.274	−0.331-	−0.310-	0.274
Ca++	−0.976**	−0.918**	0.865**	−0.961**	−0.918**	−0.980**	−0.972**	−0.941**	−0.920**	−0.025	0.209		−0.307	0.979**	0.979**	0.971**	−0.987**	−0.930**	−0.935**	0.952**
Adrenaline	0.199	0.24	−0.056	0.163	0.24	0.208	0.268	0.166	0.142	0.211	0.016	−0.307		−0.328	−0.265	−0.191	0.274	0.13	0.137	−0.216-
Catalase	−0.977**	−0.939-**	0.863**	−0.959**	−0.939**	−0.969-**	−0.966**	−0.962**	−0.905**	0.006	0.26	0.979**	−0.328		0.963**	0.975**	−0.985**	−0.925**	−0.921**	0.934**
SOD	−0.968**	−0.878-**	0.844**	−0.950**	−0.878**	−0.985-**	−0.980**	−0.943**	−0.928**	−0.009	0.206	0.979**	−0.265	0.963**		0.957**	−0.979**	−0.960**	−0.941**	0.942**
GST	−0.972**	−0.959**	0.878**	−0.952**	−0.959**	−0.976**	−0.943**	−0.968**	−0.910**	−0.033	0.243	0.971**	−0.191	0.975**	0.957**		−0.990**	−0.915**	−0.922**	0.926**
MDA	0.983**	0.945**	−0.891**	0.966**	0.945**	0.987**	0.962**	0.967**	0.931**	0.034	−0.274	−0.987**	0.274	−0.985**	−0.979**	−0.990**		0.941**	0.945**	−0.946**
TROPONIN	0.963**	0.820**	−0.894**	0.967**	0.820**	0.951**	0.943**	0.925**	0.967**	−0.150-	−0.331	−0.930**	0.13	−0.925**	−0.960**	−0.915**	0.941**		0.971**	−0.958**
CK	0.973**	0.839**	−0.946**	0.986**	0.839**	0.957**	0.919**	0.929**	0.982**	−0.070	−0.310	−0.935**	0.137	−0.921**	−0.941**	−0.922**	0.945**	0.971**		−0.925**
VIT D	−0.958**	−0.868**	0.861**	−0.952**	−0.868**	−0.939**	−0.946**	−0.888**	−0.924**	0.211	0.274	0.952**	−0.216	.934**	0.942**	0.926**	−0.946**	−0.958**	−0.925**	

**T.cholesterol, Total cholesterol; TG, Triglycerides; HDL, High Density Lipoprotein; LDL, Low Density Lipoprotein; VLDL, Very Low Density Lipoprotein; ALT, Alanine Aminotransferase; AST, Aspartate Aminotransferase; ALP, Alkaline phosphatase; T. Bilirubin, Total Bilirubin; D. Bilirubin, direct Bilirubin; TP, Total Protein; Ca+, Calcium ion; SOD, Superoxide dismutase; MDA, Malondialdehyde; CK, Creatinine Kinase.

### 3.9 Histopathology of the liver

**FIGURE 6:**
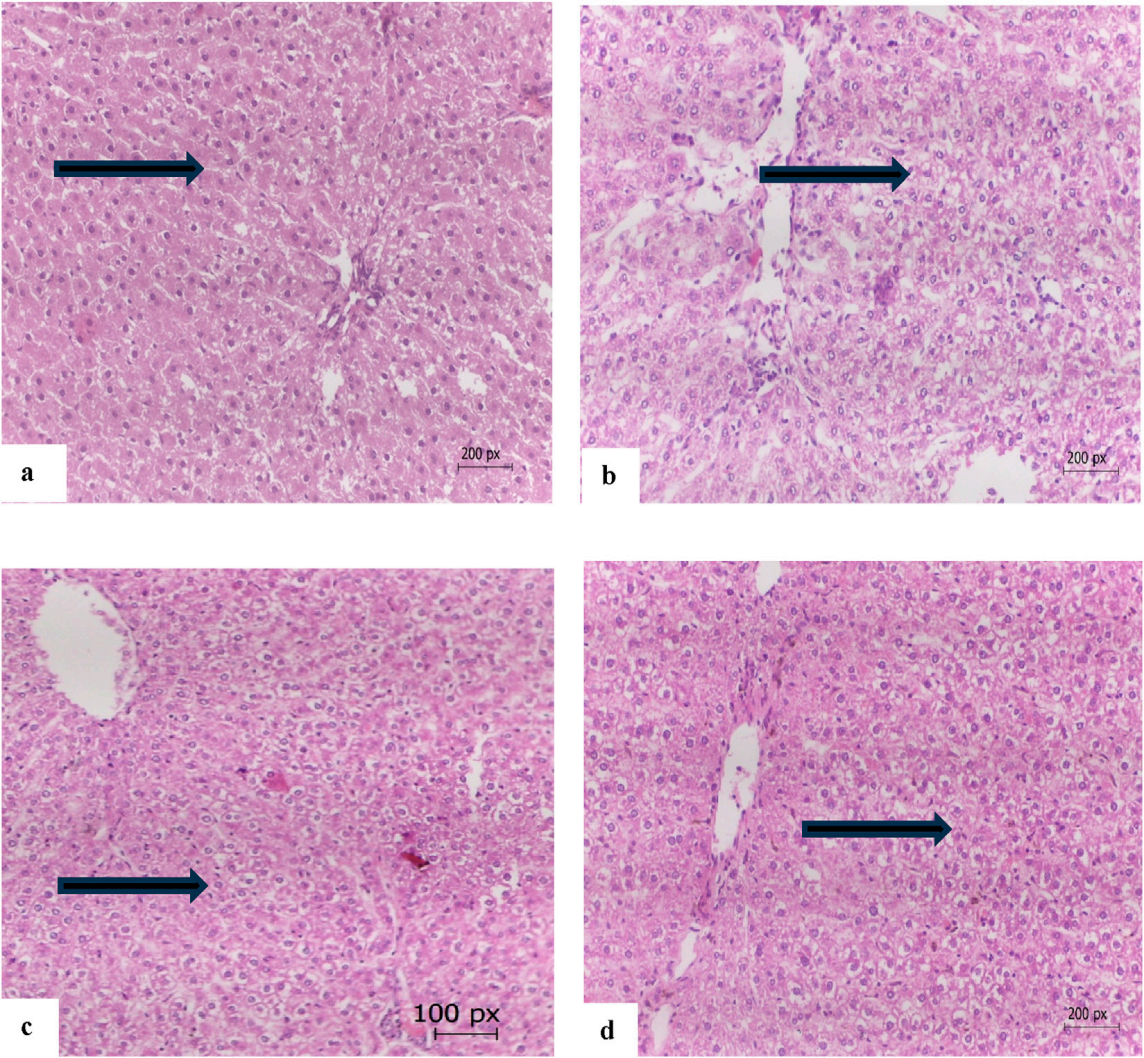
**(a)** The hepatic tissue of the control negative group (G1) showing normal hepatocytes (arrow), **(b)** the hepatic tissue of the hypercholesterolemic control positive group (G2) showing build up of fats within hepatic cells and inflammation (arrow), **(c)** hepatic tissue of the safflower-treated group (G3) showing nearly normal hepatocytes (arrow), **(d)** the hepatic tissue of atorvastatin-treated group (G4) with normal hepatocytes (arrow) (E and H 200 x).

Figure 6 illustrates the therapeutic effect of safflower and atorvastatin on hypercholesterolemia. Figure 4A presents the normal healthy hepatic tissue of the negative control group (G1), which contains normal hepatocytes. Figure 6B depicts the hypercholesterolemic hepatic tissue of the control positive group (G2), showing buildup of fat within hepatic cells, ballooning, disturbed plates, inflammation, cell damage, hepatic steatosis, and portal tract inflammation with no fibrosis. Figure 6C, treated with safflower (G3), shows that hepatic tissues restored their normal histology, exhibiting no signs of inflammation. Figure 6D shows hepatic tissue with restored normal hepatocytes subjected to atorvastatin treatment in the fourth group.

### 3.10 Histopathology of the heart

**FIGURE 7:**
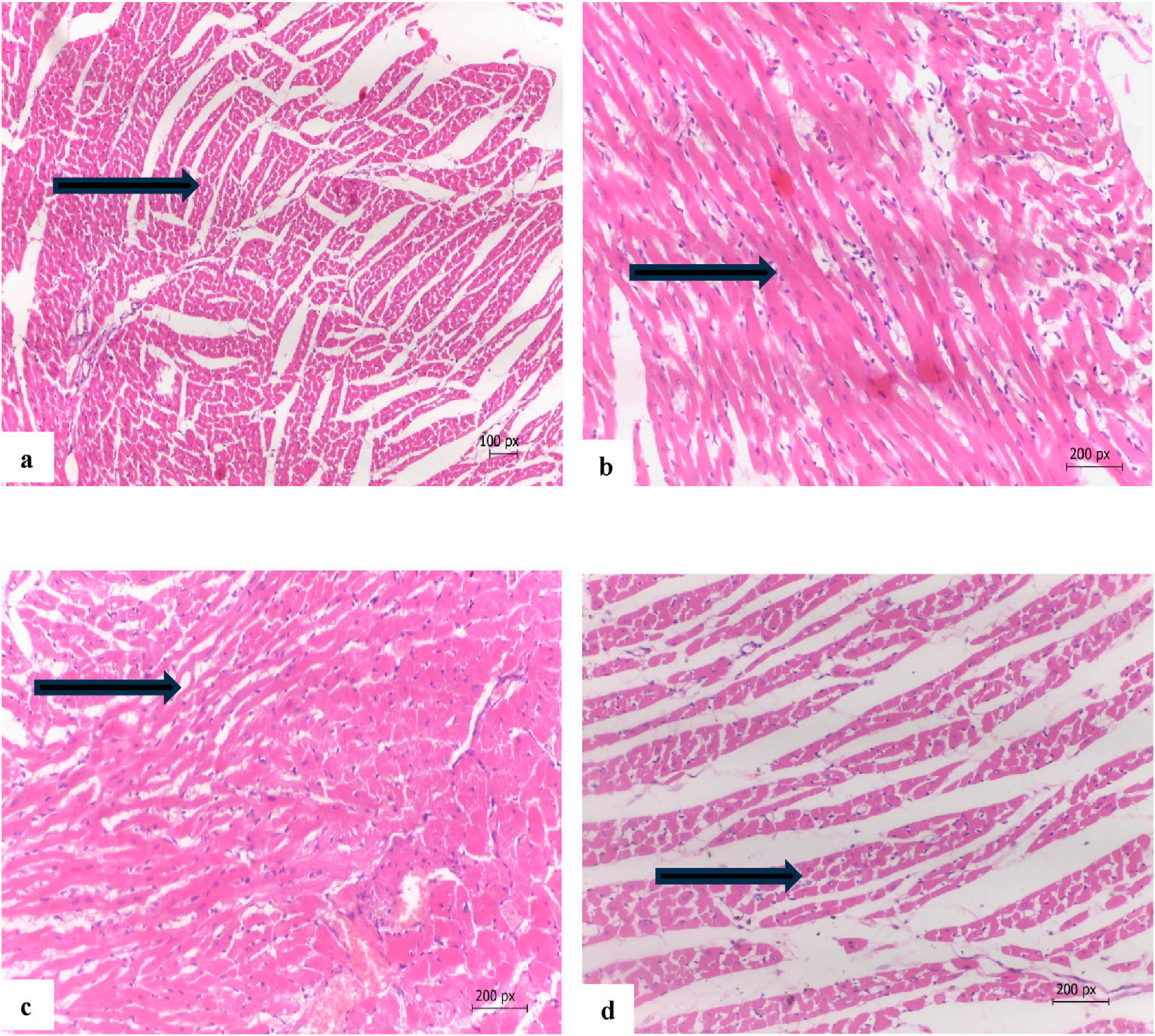
**(a)** Cardia tissue of G1 showing normal cardiac myocytes (arrow), **(b)** cardiac tissue of the hypercholesterolemic positive control (G2) myocyte atrophy, accumulation of fats between cells and fibrosis with focal myocitis (arrow), **(c)** cardiac tissue of G3 treated with safflower showing mild to moderate myocyte atrophy, fibrosis with focal myocitis (arrow), **(d)** cardiac tissue of G4 treated with atorvastatin showing nearly normal cardiac tissues with a very mild myocyte atrophy with no fibrosis or myocitis (arrow). (Hand E x200).

Figure 7 illustrates the therapeutic effect of safflower extract on the histology of cardiac tissues. Figure 7a illustrates the normal cardiac tissues of the negative control (G1), which exhibit typical cardiac myocytes without signs of edema, necrosis, or myositis. Figure 7b illustrates the cardiac tissue of the positive hypercholesterolemic group (G2), which exhibits myocyte atrophy, increased fat accumulation, inflammation, fibrosis, and focal fibrotic myocitis. Treatment of hypercholesterolemic rats in G3 with safflower resulted in reduced histopathological damage to cardiac tissues, characterized by mild to moderate myocyte atrophy and mild fibrosis with focal myocitis, as illustrated in Figure 7c. In contrast, treatment with atorvastatin in G4 healed cardiac tissues, exhibiting only very mild myocyte atrophy without fibrosis or myocitis, as depicted in Figure 7d.

### 3.11 The total body weight

The total body weight was significantly (*P* < 0.01) increased as a result of the induced hypercholesterolemia in the positive control group (G2), compared to the negative control (G1), as shown in Table 3. Safflower extract and atorvastatin administration in G3 and G4 significantly (*P* < 0.01) decreased the total body weight compared to the positive control. Atorvastatin was more effective than safflower extract in reducing the total body weight.

**TABLE 3:** Effect of treating hypercholesterolemic rats with safflower and atorvastatin for 4 weeks on total body weight (g).

Variables	Statistics	G1 −ve control	G2 +ve control	G3 Safflower	G4 Atorvastatin
Initial weight (g)	Mean ± SE LSD (0.05) = 1.97 t-test	182.3 ± 0.6^a^	182.3 ± 0.9^a^ -	182.3 ± 0.6^a^ -	183.7 ± 0.6^a^ −1.69 ^NS^
1st week (g)	Mean ± SE LSD (0.05) = 1.99 t-test	186.3 ± 0.6^a^	187.0 ± 0.4^a^ −1.00 ^NS^	186.3 ± 0.9^a^ -	186.0 ± 0.7^a^ 0.36 ^NS^
2nd week (g)	Mean ± SE LSD (0.05) = 1.62 t-test	190.7 ± 0.4^c^	193.7 ± 0.6^b^ −4.29**	191.3 ± 0.4^c^ −1.12 ^NS^	190.0 ± 0.7^c^ 0.79 ^NS^
3rd week (g)	Mean ± SE LSD (0.05) = 1.62 t-test	194.0 ± 0.4^c^	200.7 ± 0.6^b^ −10.00***	196.3 ± 0.4^c^ −4.18 **	195.3 ± 0.8^c^ −1.58 ^NS^
4th week (g)	Mean ± SE LSD (0.05) = 1.67 t-test	199.0 ± 0.6^c^	209.3 ± 0.4^b^ −13.59***	204.0 ± 0.4^d^ −6.85 ***	200.3 ± 0.8^c^ −1.35 ^NS^

Data are represented as Mean ± SE. t-test value “∗∗∗” means highly significant at *P* < 0.001, t-test value “∗∗” means significant at *P* < 0.01, NS means non-significant. ANOVA analysis within groups: means with different superscripts (a, b, c, or d) show significant difference at *P* < 0.05, while means superscripts with the same letters mean that there is no significant difference at *P* < 0.05. LSD: Least Significant Difference.

## 4 Discussion

This study evaluated the therapeutic advantages of *C. tinctorius aqueous* extract and atorvastatin in male rats with induced hypercholesterolemia. This investigation utilized the aqueous extract of safflower flowers, a prevalent food ingredient, and the trial was limited to 4 weeks to mitigate the risk of toxicity associated with prolonged safflower administration (Adamska and Biernacka, 2021; Alshareef et al., 2024).

Administration of a fat-rich food to the experimental animals resulted in hypercholesterolemia in the positive control group, as evidenced by increased triglycerides, total cholesterol, LDL, and vLDL, alongside a reduction in HDL (El Rabey et al., 2018; Alshareef et al., 2024). The administration of safflower and atorvastatin significantly restored the lipid profile of the hypercholesterolemic rats in G3 and G4, respectively. *C. tinctorius* has various health advantages, including antioxidant, antidiabetic, anti-inflammatory, and analgesic properties, in addition to its nutritional benefits (Che et al., 2016). These significant therapeutic benefits can be attributed to its higher content of polyphenols, such as quercetin, chlorogenic acid, and HSYA’s (Adamska and Biernacka, 2021). The polyphenols of safflower, such as quercetin and chlorogenic acid, and HSYA’s enhance the production and activity of hormone-sensitive lipase (HSL) while inhibiting adipocyte proliferation (Sandoval et al., 2020). Conversely, atorvastatin is an approved drug for lowering cholesterol levels and preventing cardiovascular diseases (Livingstone et al., 2016; Abd-Elmonsif and Gamal, 2025). It reduces cholesterol by inhibiting 3-hydroxy-3-methylglutaryl-coenzyme A (HMG-CoA) reductase, thereby preventing the conversion of HMG-CoA to mevalonate, which diminishes cholesterol synthesis in the liver and enhances the quantity of LDL receptors on hepatic cells (McIver and Siddique, 2025; Dagli-Hernandez et al., 2022; Abd-Elmonsif and Gamal, 2025).

The induction of hypercholesterolemia in the positive control group markedly impacted liver function, elevating liver enzymes and bilirubin levels while diminishing total protein (Alshareef et al., 2024). Furthermore, these modified liver functions resulted from the direct manifestation of the histopathological damage caused by hypercholesterolemia on the liver tissues (Al-Sieni et al., 2020). The hepatoprotective effect of safflower in the treatment group may be attributed to the free radical scavenging properties of its polyphenolic compounds (Ruyvaran et al., 2022; Alshareef et al., 2024). Moreover, atorvastatin’s protective effects may stem from its essential function in inhibiting fat buildup, hence enhancing the functionality of other organs and mitigating cardiovascular illnesses and dyslipidemia (Livingstone et al., 2016; Abd-Elmonsif and Gamal, 2025). Safflower also protected against liver damage and hepatic steatosis in type 2 diabetes mellitus via Nrf2-dependent pathways, attributable to its antioxidant, hypoglycemic, hypolipidemic, and anti-inflammatory properties (Alshareef et al., 2024).

The induction of hypercholesterolemia resulted in increased lipid peroxidation, as indicated by elevated MDA levels and reduced activity of the antioxidant enzymes catalase, SOD, and GSH, in comparison to the negative control group. Conversely, treatment with safflower and atorvastatin improved these disrupted oxidative stress parameters. Furthermore, the hepatoprotective properties of safflower may be attributed to polyphenols such as quercetin, chlorogenic acid, and HSYA’s ability to augment the synthesis of hepatic antioxidative enzymes that regulate obesity. Additionally, the administration of safflower extract, which comprises both HSYA and safflower yellow (also known as carthamine yellow), elevates the mRNA levels of antioxidative enzymes and enhances the superoxide dismutase (SOD) activity in liver homogenates (Yan et al., 2020). HSYA addresses obesity by modifying intestinal microbiota, enhancing particular digestive bacterial populations in the colon, and optimizing digestive tract function and systemic metabolism (Adamska and Biernacka, 2021).

The elevation of heart function enzyme activity (troponin and CK) in the hypercholesterolemic positive control group is corroborated by histological analyses that indicated damaged cardiac tissues and oxidative stress markers (El Rabey et al., 2018; Al-Sieni et al., 2020). The notable enhancement in cardiac function metrics following treatment with safflower and atorvastatin may be attributed to their hypolipidemic and antioxidant properties, which alleviated oxidative stress (Yan et al., 2020; Abd-Elmonsif and Gamal, 2025). Recent studies have identified hyperlipidemia as a risk factor for stroke in patients with coronary vascular diseases (CVD), prompting ongoing research aimed at mitigating this risk factor to reduce the incidence of stroke and myocardial infarction (MI) (Kim et al., 2017; Olamoyegun et al., 2016; Alloubani et al., 2021).

The current findings indicated a notable reduction in vitamin D and calcium levels due to induced hypercholesterolemia in the positive control group, while treatment with safflower and atorvastatin resulted in an elevation of these levels. Calcium and vitamin D levels exhibit a positive correlation; therefore, calcium is essential for vitamin D production. Recent research indicates that hypercholesterolemia is associated with a deficiency in vitamin D levels and *vice versa* (Dibaba, 2019; Han et al., 2021). The elevation of calcium levels is associated with the enhancement of vitamin D to optimal levels; thus, vitamin D is integral to calcium homeostasis at these optimal levels (Mattioli et al., 2025). Furthermore, Che et al. (2016) indicated that safflower seeds exhibit protective effects against bone loss induced by estrogen shortage through the stimulatory action of polyphenolic chemicals on osteoblast growth. Vitamin D facilitates the development of healthy calcified vertebrate skeletons, whereas its receptor in calcium-regulating organs modulates calcium metabolism and enhances bone health (Izzo et al., 2024). In addition, liver illness is associated with a deficit in vitamin D (Elangovan et al., 2017). Vitamin D supplementation safeguards liver health from fibrosis, and its production is contingent upon the liver’s health and functionality (Keane et al., 2018).

The induced hypercholesterolemia markedly elevated adrenaline levels in the present investigation. This finding is consistent with earlier research linking hypercholesterolemia to elevated adrenaline levels (Maduka et al., 2015; Fulton et al., 2022). Treatment of hypercholesterolemic rats with safflower and atorvastatin enhanced adrenaline levels, practically returning them to normal as observed in the negative control group. This enhancement is associated with the hypolipidemic effects of safflower and atorvastatin, which benefit the liver. Adrenaline is a catecholamine, along with dopamine and norepinephrine, that significantly contributes to the progression of liver disorders (Lelou et al., 2022). The preventive impact of safflower may be attributed to its elevated levels of the polyphenol’s quercetin and chlorogenic acid, recognized for their superior antioxidant, antidiabetic, and hypolipidemic properties (Jiang et al., 2019; El Rabey et al., 2018; Liu et al., 2022). The association of vitamin D with hypercholesterolemia and the adrenal gland is attributed to their shared genetic pathway (Al Refaie et al., 2022).

The induced hypercholesterolemia also caused hepatic and cardiac histological alterations in the positive control group relative to the negative control (El Rabey et al., 2018; Al-Sieni et al., 2020). These altered changes in the hepatic and cardiac tissues caused an elevation in the liver and heart markers in the positive control group (Lelou et al., 2022; Ognik et al., 2021). Administration of safflower and atorvastatin to hypercholesterolemic rats restored the histology of liver and heart, approaching the normal state and protecting against histological alterations (Lelou et al., 2022; Liu et al., 2022).

The total body weight was gradually increased with increasing hypercholesterolemia (Al-Sieni et al., 2020; El Rabey et al., 2020; Liu et al., 2022), while treating these hypercholesterolemic rats with safflower and atorvastatin significantly ceased this weight gain. The quercetin (an Omega-3 fatty acid) and chlorogenic acid, which are naturally occurring phenolic compounds, represent the major constituents of safflower, which play a significant role in improving lipid metabolism and thus decreasing weight gain compared to the positive control group (Jiang et al., 2019; Tahir et al., 2023). Similarly, atorvastatin treated the hypercholesterolemia and thus attenuated the increased body weight gain (Livingstone et al., 2016; Abd-Elmonsif and Gamal, 2025).

Atorvastatin is an effective cholesterol-lowering medication recommended by the FDA (Livingstone et al., 2016; Abd-Elmonsif and Gamal, 2025). In this study, we observed that treatment with safflower increased antioxidant enzyme activity, calcium, and vitamin D, and decreased total cholesterol, LDL, triglycerides, lipid peroxidation, liver function enzymes, adrenaline, troponin, and CK levels (Fulton et al., 2022; Alshareef et al., 2024; Deepika et al., 2025).

Safflower extract has been used in folk medicine for short periods and in specific doses to avoid toxicity resulting from large doses. It is used to treat many diseases such as hyperlipidemia, diabetes, and many symptoms associated with anxiety, stress, and fear. Hence, the idea for this study arose, and we hope that the results of this study will be used to develop pharmaceutical formulations of safflower to treat these diseases and alleviate pain. The dosage of safflower extract may need to be elevated to achieve enhanced protective efficacy. Additional research is necessary to investigate the function of each component in improving health and elucidating its protective effects on different organs. In addition, this study was conducted on rats, despite they do not accurately model human lipoprotein profiles or atherosclerosis progression, because rodents are considered ideal for atherosclerosis research because of their fast breeding, low cost of production, and short time needed for developing atherosclerosis (Daniels et al., 2018; Zhao et al., 2020).

Safflower and atorvastatin exhibited numerous health advantages. They elevated antioxidant and vitamin D levels and reduced hyperlipidemia, lipid peroxidation, CK, troponin, and adrenaline. They further safeguarded the liver and heart of the generated hypercholesterolemic animals. They also function as anxiolytics by reducing adrenaline levels. Atorvastatin has shown superior efficacy compared to safflower in safeguarding against hypercholesterolemia and its associated consequences.

## Data Availability

The original contributions presented in the study are included in the article/Supplementary Material, further inquiries can be directed to the corresponding author.

## Supplementary material

SUPPLEMENTARY FIGURE 1 Polyphenols of safflower aqueous extract.

## Abbreviations

ADR, Adverse drug reaction; ALP, alkaline phosphatase; ALT, Alanine aminotransferase; ANOVA, analysis of variance; AST, aspartate transaminase; Cat, Catalase; Cat, catalase; CK, creatine kinase; CK, Creatine kinase; GST, glutathione-s-transferase; GST, glutathione-s-transferase; HDL, high-density lipoprotein; HSYA, hydroxysafflor; IFCC, International Federation of Clinical Chemistry and Laboratory Medicine; LDL, low-density lipoproteins; LSD, least significant difference; MDA, malondialdehyde; MDA, malondialdehyde; SOD, super oxide dismutase; TC, total cholesterol; TG, triglycerides; TP, total protein; vLDL, very low-density lipoproteins.
