# Supplementary material for: Comparison between the protective effect of the orally administered atorvastatin and safflower (Carthamus tinctorius) in hypercholesterolemic male rats
Source: Front Pharmacol. 2025 Sep 15;16:1663717. doi: 10.3389/fphar.2025.1663717 (PMC12477430; doi:10.3389/fphar.2025.1663717)
Supplement: Supplementary file 1 [file Table1.docx]

**Supplementary Table 1.** Effect of treating rats with safflower and atorvastatin on antioxidant enzymes and lipid peroxidation in induced hypercholesterolemic male rats.

|  | **Statistics** | **G1**  **−ve control** | **G2**  **+ve control** | **G3 Safflower** | **G4**  **Ator** |
| --- | --- | --- | --- | --- | --- |
| **SOD**  **U/L** | Mean± SE  LSD (0.05)=8.11  t-test | 324.0±2.6 ^a^ | 124.0±2.9 ^c^  51.76*** | 185.7±1.6 ^d^  45.50 *** | 271.7±3.5 ^b^  11.97 *** |
| **Catalase**  **U/L** | Mean± SE  LSD (0.05)=0.26  t-test | 4.4±0.11 ^b^ | 0.6±0.03 ^c^  33.16*** | 1.6±0.08 ^d^  20.71 *** | 2.7±0.10 ^a^  11.35 *** |
| **GST**  **U/L** | Mean± SE  LSD (0.05)=8.99  t-test | 289.0±1.3 ^c^ | 118.0±2.0 ^d^  70.60*** | 165.3±4.8 ^b^  25.01 *** | 201.7±2.9 ^a^  27.20 *** |
| **MDA**  **nmole/1 ml** | Mean± SE  LSD (0.05)=0.42  t-test | 1.1±0.02 ^a^ | 14.9±0.11 ^d^  -123.79*** | 10.7±0.13 ^b^  -71.98 *** | 7.1±0.22 ^c^  -26.58 *** |

SOD: superoxide dismutase, GST: Glutathione-s-transferase, MDA: Malondialdehyde.

Data are represented as Mean ± SE. t-test value “∗∗∗” means highly significant at 𝑃 < 0.001. ANOVA analysis within groups: means with different superscripts (a, b, c, or d) show significant difference at 𝑃 < 0.05, while means superscripts with the same letters mean that there is no significant difference at 𝑃 < 0.05. LSD: Least Significant Difference.
