# Supplementary material for: Comparison between the protective effect of the orally administered atorvastatin and safflower (Carthamus tinctorius) in hypercholesterolemic male rats
Source: Front Pharmacol. 2025 Sep 15;16:1663717. doi: 10.3389/fphar.2025.1663717 (PMC12477430; doi:10.3389/fphar.2025.1663717)
Supplement: Supplementary file 2 [file Table2.docx]

**Supplementary Table 2.** The hypolipidemic effect of safflower and atorvastatin administration on liver function parameters in induced hypercholesterolemic male rats.

|  | **Statistics** | **G1**  **−ve control** | **G2**  **+ve control** | **G3 Safflower** | **G4**  **Atorvastatin** |
| --- | --- | --- | --- | --- | --- |
| ALT  U/L | Mean± SE  LSD (0.05)=3.61  t-test | 18.0±0.7 ^a^ | 82.0±0.7 ^d^  -61.97*** | 61.0±1.5 ^b^  -26.33*** | 41.0±1.7 ^c^  -12.60 *** |
| AST  U/L | Mean± SE  LSD (0.05)=4.72  t-test | 20.7±1.6 ^a^ | 81.0±1.3 ^d^  -28.62*** | 66.3±1.3 ^b^  -21.88 | 38.0±2.0 ^c^  -6.63 *** |
| ALP  U/L | Mean± SE  LSD (0.05)=6.47  t-test | 107.0±3.2 ^a^ | 158.0±2.4 ^d^  -12.65*** | 140.0±1.5 ^b^  -9.27*** | 127.3±0.9 ^c^  -6.83 ** |
| T.Bilirubin  mg/dl | Mean± SE  LSD (0.05)=0.11  t-test | 0.43±0.01 ^a^ | 1.50±0.07 ^d^  -14.56*** | 0.90±0.01 ^b^  -50.78*** | 0.68±0.01 ^c^  -26.52 *** |
| D.Bilirubin  mg/dl | Mean± SE  LSD (0.05)=0.04  t-test | 0.23±0.01 ^a^ | 0.22±0.01 ^a^  0.22^NS^ | 0.24±0.02 ^a^  -0.56 ^NS^ | 0.23±0.01 ^a^  -0.25 ^NS^ |
| TP  gm/dl | Mean± SE  LSD (0.05)=0.39  t-test | 6.33±0.08 ^a^ | 6.01±0.24 ^c^  1.32 ^NS^ | 6.43±0.08 ^a^  -0.88 ^NS^ | 6.22±0.06 ^a^  1.18 ^NS^ |

ALT: Alanine transaminase, AST: Aspartate transferase, ALP: alkaline phosphatase, T. bilirubin: Total bilirubin, D. bilirubin: Direct bilirubin, TP: Total protein.

Data are represented as Mean ± SE. t-test value “∗∗∗” means highly significant at 𝑃 < 0.001. ANOVA analysis within groups: means with different superscripts (a, b, c, or d) show significant difference at 𝑃 < 0.05, while means superscripts with the same letters mean that there is no significant difference at 𝑃 < 0.05. LSD: Least Significant Difference.
