# Supplementary material for: Comparison between the protective effect of the orally administered atorvastatin and safflower (Carthamus tinctorius) in hypercholesterolemic male rats
Source: Front Pharmacol. 2025 Sep 15;16:1663717. doi: 10.3389/fphar.2025.1663717 (PMC12477430; doi:10.3389/fphar.2025.1663717)
Supplement: Supplementary file 3 [file Table3.docx]

**Supplementary Table 3.** Effect of treating induced hypercholesterolemia rats with Safflower and atorvastatin for 4 weeks on serum cardiac enzymes (CK and Troponin), Ca^++^, vitamin D, and adrenaline.

|  | **Statistics** | **G1**  **−ve control** | **G2**  **+ve control** | **G3 Safflower** | **G4**  **Ator** |
| --- | --- | --- | --- | --- | --- |
| **TROPONIN**  **ng/mL** | Mean± SE  LSD (0.05)=0.18  t-test | 0.4±0.01 ^a^ | 2.9±0.02 ^d^  -128.69*** | 1.6±0.11 ^b^  -10.21 *** | 0.9±0.03 ^c^  -14.38 *** |
| **CK**  **U/L** | Mean± SE  LSD (0.05)=10.57  t-test | 161.0±1.0 ^a^ | 384.3±3.9 ^d^  -55.53*** | 256.7±2.5 ^b^  -35.88 *** | 223.7±5.4 ^c^  -11.46 *** |
| **Ca^++^**  **mmol/L** | Mean± SE  LSD (0.05)=0.30  t-test | 11.3±0.09 ^b^ | 7.1±0.03 ^c^  43.21*** | 8.3±0.14 ^d^  18.07 *** | 9.7±0.11 ^a^  11.07 *** |
| **Vit D**  **ng/mL** | Mean± SE  LSD (0.05)= 2.48  t-test | 28.1±1.1 ^b^ | 11.1±0.5 ^c^  14.13*** | 17.5±0.9 ^d^  7.55 *** | 22.2±0.8 ^a^  4.30 ** |
| **Adrenaline**  **pg/mL** | Mean± SE  LSD (0.05)=85.37  t-test | 10.9±0.1 ^a^ | 37.9±1.7 ^d^  -15.67*** | 27.8±0.8 ^b^  -21.44 *** | 18.7±0.8 ^c^  -10.08 *** |

CK: Creatine kinase, Vit D: vitamin D.

Data are represented as Mean ± SE. t-test value “∗∗∗” means highly significant at 𝑃 < 0.001 and t-test value “∗∗” means significant at 𝑃 < 0.01. ANOVA analysis within groups: means with different superscripts (a, b, c, or d) show significant difference at 𝑃 < 0.05, while means superscripts with the same letters mean that there is no significant difference at 𝑃 < 0.05. LSD: Least Significant Difference.
