# Supplementary material for: Comparison between the protective effect of the orally administered atorvastatin and safflower (Carthamus tinctorius) in hypercholesterolemic male rats
Source: Front Pharmacol. 2025 Sep 15;16:1663717. doi: 10.3389/fphar.2025.1663717 (PMC12477430; doi:10.3389/fphar.2025.1663717)
Supplement: Supplementary file 4 [file Image1.pdf]

===

Acq. Operator : SYSTEM Seq. Line : 1
Acq. Instrument : hplc -2 Location : Vial 1
Injection Date : 10/8/2024 4:09:17 PM Inj : 1
Inj Volume : 5.000 µl
Acq. Method : C:\CHEM32\1\DATA\PP 8-10-2024 2024-10-08 16-07-33\POLYPHENOL 2023.M
Last changed : 10/8/2024 4:07:36 PM by SYSTEM
Analysis Method : C:\CHEM32\1\DATA\PP 8-10-2024 2024-10-08 16-07-33\POLYPHENOL 2023.M (Sequence Method)
Last changed : 10/9/2024 10:20:58 AM by SYSTEM
(modified after loading)
Additional Info : Peak(s) manually integrated

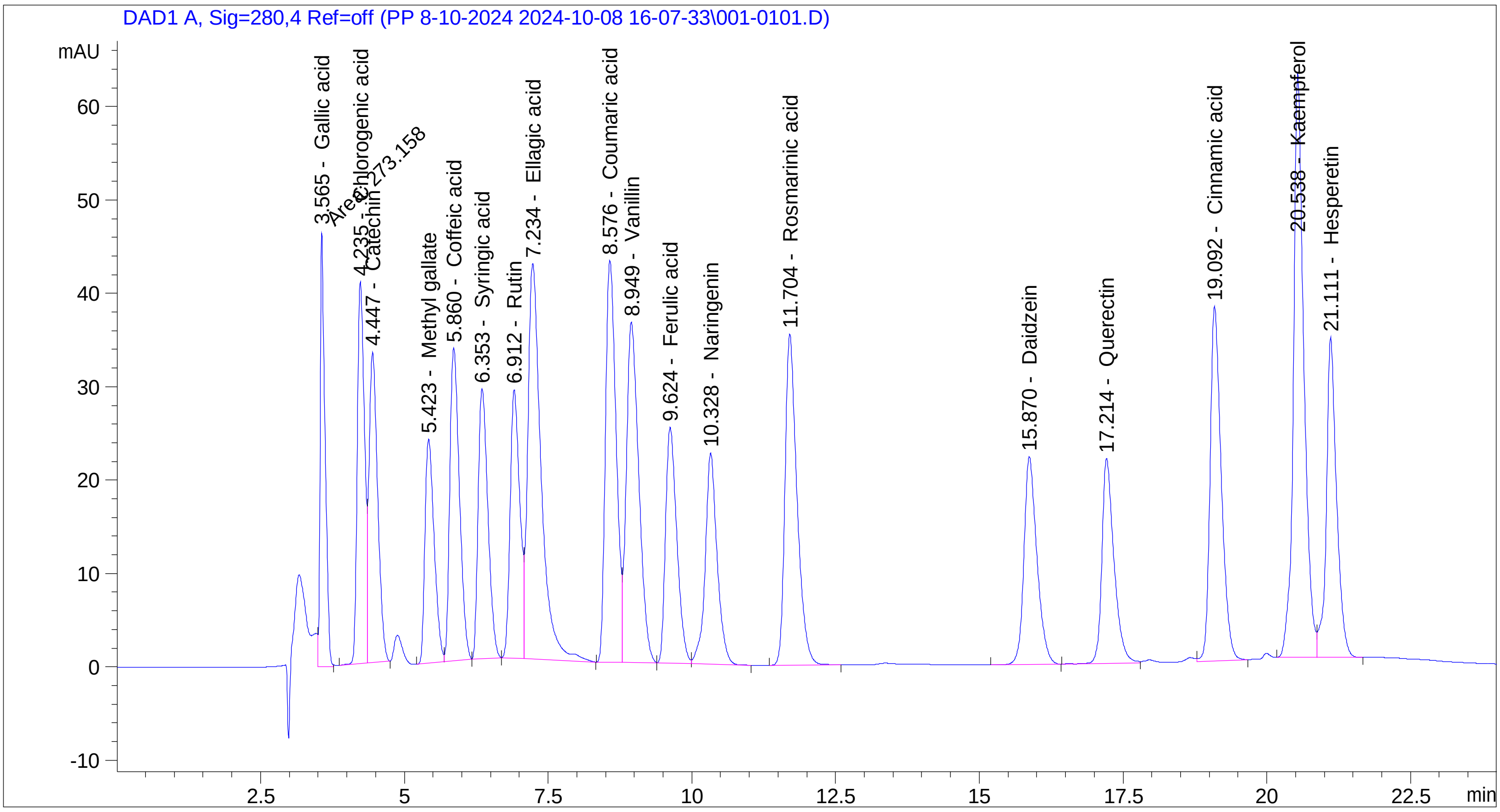

===

Area Percent Report

===

Sorted By : Signal
Calib. Data Modified : 10/9/2024 10:20:57 AM
Multiplier : 1.0000
Dilution : 1.0000
Do not use Multiplier & Dilution Factor with ISTDs

Signal 1: DAD1 A, Sig=280,4 Ref=off

Peak #	RetTime [min]	Type	Width [min]	Area [mAU*s]	Area %	Name
1	3.565	MM	0.0974	273.15796	3.5459	Gallic acid
2	4.235	BV	0.1383	358.81638	4.6578	Chlorogenic acid
3	4.447	VB	0.1584	349.11911	4.5319	Catechin
4	5.423	BV	0.1742	268.08408	3.4800	Methyl gallate
5	5.860	VB	0.1817	389.83694	5.0605	Coffeic acid

Peak #	RetTime [min]	Type	Width [min]	Area [mAU*s]	Area %	Name
6	6.353	BB	0.1832	340.02362	4.4139	Syringic acid
7	6.912	BV	0.1754	334.01620	4.3359	Rutin
8	7.234	VB	0.2418	689.06519	8.9448	Ellagic acid
9	8.576	BV	0.2028	556.48816	7.2238	Coumaric acid
10	8.949	VB	0.2326	551.42828	7.1581	Vanillin
11	9.624	BV	0.2112	344.53088	4.4724	Ferulic acid
12	10.328	VB	0.2115	325.04919	4.2195	Naringenin
13	11.704	BB	0.2153	514.82947	6.6830	Rosmarinic acid
14	15.870	BB	0.2349	349.29749	4.5342	Daidzein
15	17.214	BV	0.2167	321.17203	4.1691	Querectin
16	19.092	VB	0.2044	515.96442	6.6977	Cinnamic acid
17	20.538	BV	0.1864	795.56091	10.3272	Kaempferol
18	21.111	VB	0.1812	427.11325	5.5444	Hesperetin

Totals : 7703.55356

19 Warnings or Errors (10 first messages follow) :

Warning : Calibration warnings (see calibration table listing)
Warning : Invalid calibration curve, (Gallic acid)
Warning : Invalid calibration curve, (Chlorogenic acid)
Warning : Invalid calibration curve, (Catechin)
Warning : Invalid calibration curve, (Methyl gallate)
Warning : Invalid calibration curve, (Coffeic acid)
Warning : Invalid calibration curve, (Syringic acid)
Warning : Invalid calibration curve, (Rutin)
Warning : Invalid calibration curve, (Ellagic acid)
Warning : Invalid calibration curve, (Coumaric acid)

==

*** End of Report ***
